# Supplementary material for: Dialectical Behaviour Therapy Improves Emotion Dysregulation Mainly in Binge Eating Disorder and Bulimia Nervosa: A Systematic Review and Meta-Analysis
Source: J Pers Med. 2021 Sep 18;11(9):931. doi: 10.3390/jpm11090931 (PMC8470932; doi:10.3390/jpm11090931)
Supplement: Supplementary file 1 [file jpm-11-00931-s001.zip › Supplementary_Materials_DBT_improves_emotion_ dysregulation_in_ED.pdf]

Supplementary Materials

for

Dialectical Behavioural Therapy Improves Emotional Dysregulation Mainly in Binge Eating Disorder and Bulimia Nervosa: A Systematic Review and Meta-analysis

Natalia Rozakou-Soumalia, Ştefana Dârvariu and Jan Magnus Sjögren

1. Supplementary figures

Meta-regression	Number of obs = 31
**REML** estimate of between-study variance	tau2 = **.09172**
% residual variation due to heterogeneity	I-squared_res = **22.74%**
Proportion of between-study variance explained	Adj R-squared = **23.31%**
**With** Knapp-Hartung modification	

SMD	Coef.	Std. Err.	t	P>|t|	[95% Conf.	Interval]
BMI	.0384911	.0221322	1.74	0.093	-.0067743	.0837565
_cons	-1.602501	.7540735	-2.13	0.042	-3.144755	-.060248

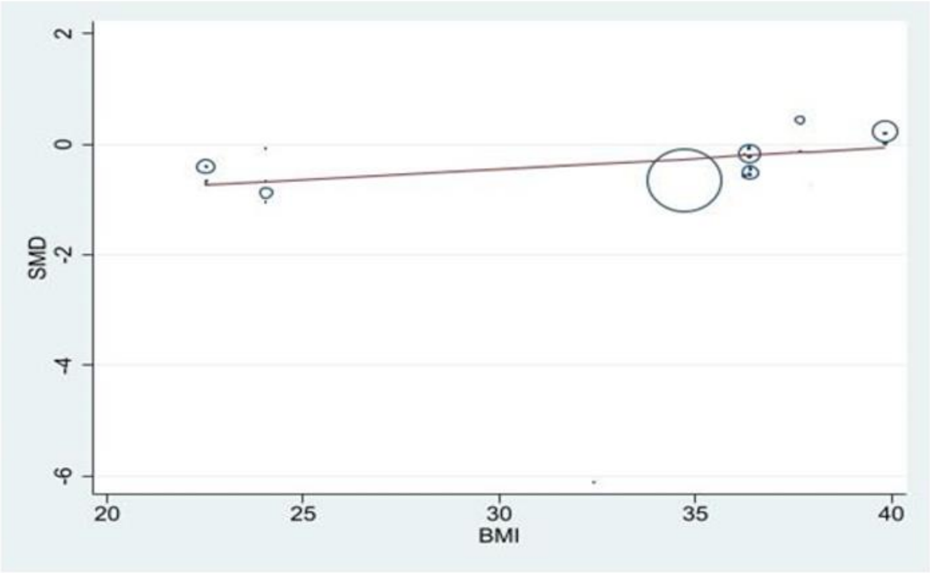

Figure S1. *Random effects meta-regression: BMI type as predictor/explanatory variable for the observed effect on emotion regulation*

```
. metareg SMD Participantnr, wsse (SE)

Meta-regression                                    Number of obs  =      36
REML estimate of between-study variance            tau2           =   .1039
% residual variation due to heterogeneity          I-squared_res  =   0.00%
Proportion of between-study variance explained     Adj R-squared  =  41.39%
With Knapp-Hartung modification
```

SMD	Coef.	Std. Err.	t	P>|t|	[95% Conf.	Interval]
Participantnr	.0077186	.0040511	1.91	0.065	-.0005142	.0159514
_cons	-.6860122	.2834549	-2.42	0.021	-1.262062	-.1099625

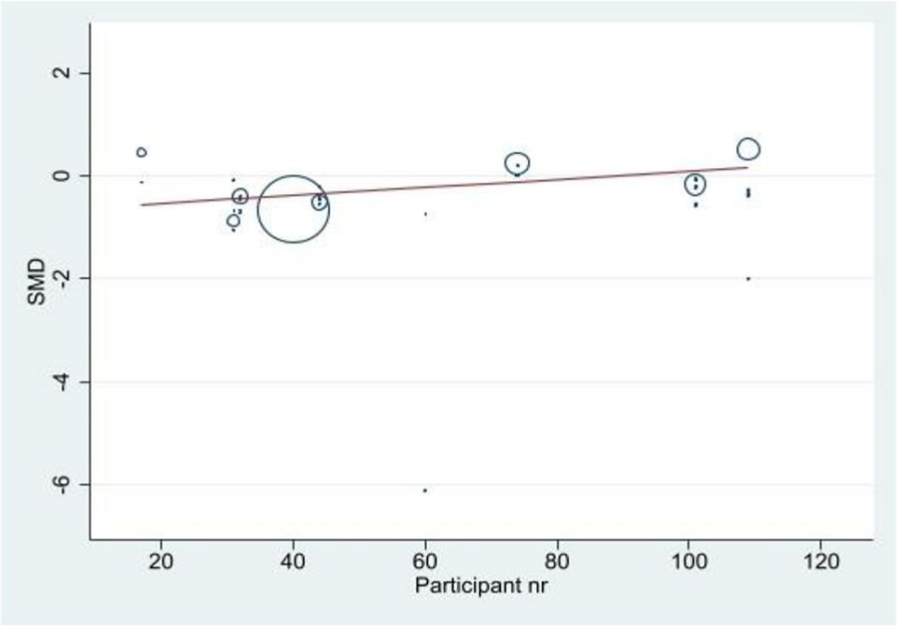

Figure S2. *Random effects meta-regression: sample size as predictor/explanatory variable for the observed effect on emotion regulation*

```
 metareg SMD Age, wsse (SE) graph

eta-regression                                      Number of obs   =        35
EML estimate of between-study variance              tau2            =     .1875
 residual variation due to heterogeneity            I-squared_res   =     0.27%
roportion of between-study variance explained       Adj R-squared   =   -15.96%
ith Knapp-Hartung modification
```

SMD	Coef.	Std. Err.	t	P>|t|	[95% Conf.	Interval]
Age	-.0004869	.0154198	-0.03	0.975	-.0318588	.030885
_cons	-.1266283	.6186108	-0.20	0.839	-1.385201	1.131945

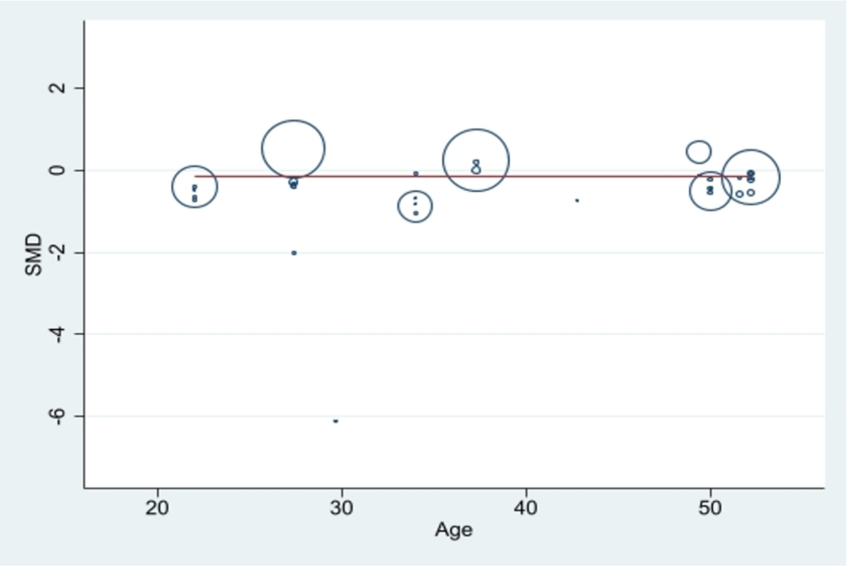

Figure S3. *Random effects meta-regression: age as predictor/explanatory variable for the observed effect on emotion regulation*

```
. metareg SMD Country, wsse (SE) graph

Meta-regression                                    Number of obs  =        36
REML estimate of between-study variance            tau2           =     .1748
% residual variation due to heterogeneity          I-squared_res  =    59.88%
Proportion of between-study variance explained     Adj R-squared  =     1.42%
With Knapp-Hartung modification
```

SMD	Coef.	Std. Err.	t	P>|t|	[95% Conf.	Interval]
Country	.3006338	.3149495	0.95	0.347	-.3394206	.9406881
_cons	-.6431704	.4775942	-1.35	0.187	-1.613759	.3274178

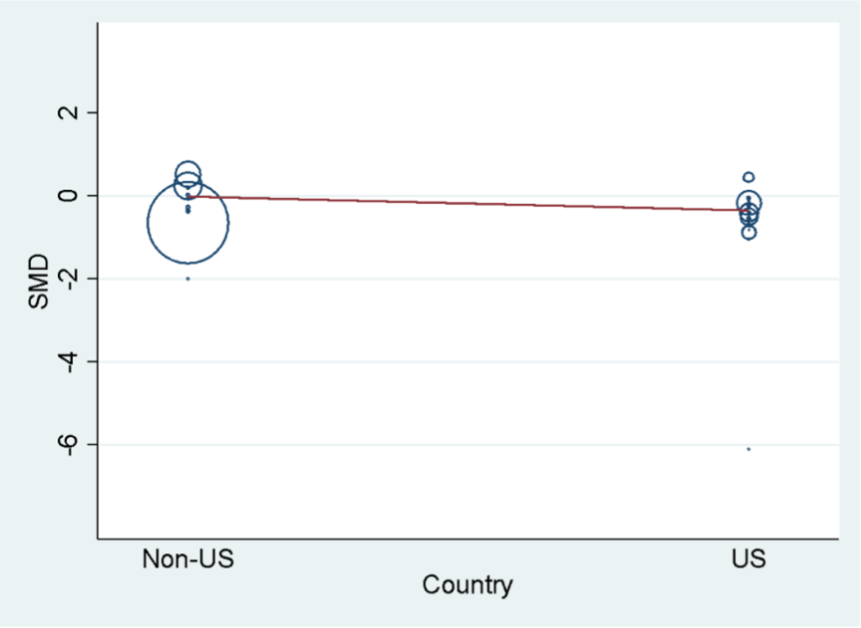

Figure S4. *Random effects meta-regression: country (US vs. non-US) as predictor/explanatory variable for the observed effect on emotion regulation*

Meta-regression | Number of obs = **36**
REML estimate of between-study variance | tau2 = **.1784**
% residual variation due to heterogeneity | I-squared_res = **39.21%**
Proportion of between-study variance explained | Adj R-squared = **-0.64%**
With Knapp-Hartung modification

SMD	Coef.	Std. Err.	t	P>|t|	[95% Conf.	Interval]
EDcode	**.1338565**	**.2112687**	**0.63**	**0.531**	**-.2954931**	**.5632061**
_cons	**-.4165565**	**.3582028**	**-1.16**	**0.253**	**-1.144512**	**.3113992**

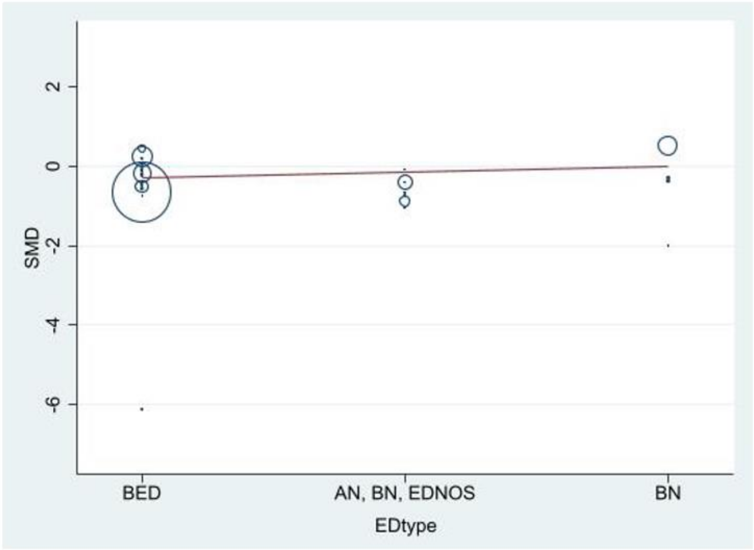

Figure S5. *Random effects meta-regression: ED type as predictor/explanatory variable for the observed effect on emotion regulation*

```
. metareg SMD Age BMI, wsse (SE) graph

Meta-regression                                     Number of obs  =        30
REML estimate of between-study variance             tau2           =   .009706
% residual variation due to heterogeneity           I-squared_res  =     0.00%
Proportion of between-study variance explained      Adj R-squared  =    91.09%
Joint test for all covariates                       Model F(2,27)  =      5.54
With Knapp-Hartung modification                     Prob > F       =    0.0096
```

SMD	Coef.	Std. Err.	t	P>|t|	[95% Conf.	Interval]
Age	-.021052	.0120397	-1.75	0.092	-.0457553	.0036513
BMI	.0637487	.0194492	3.28	0.003	.0238421	.1036553
_cons	-1.48944	.5050421	-2.95	0.007	-2.525701	-.4531794

Figure S6. *Random effects meta-regression: Combination of age & BMI as predictor/explanatory variable for the observed effect on emotion regulation*

```
. metareg SMD Age BMI Participantnr, wsse (SE) graph

Meta-regression                                     Number of obs  =        30
REML estimate of between-study variance             tau2           =    .04325
% residual variation due to heterogeneity           I-squared_res  =     0.00%
Proportion of between-study variance explained      Adj R-squared  =    60.28%
Joint test for all covariates                       Model F(3,26)  =      2.28
With Knapp-Hartung modification                     Prob > F       =    0.1029
```

SMD	Coef.	Std. Err.	t	P>|t|	[95% Conf.	Interval]
Age	-.0209287	.0163006	-1.28	0.210	-.0544351	.0125776
BMI	.0667679	.0277627	2.40	0.024	.0097008	.123835
Participantnr	-.0007043	.0052514	-0.13	0.894	-.0114988	.0100901
_cons	-1.555403	.6380935	-2.44	0.022	-2.867023	-.2437825

Figure S7. *Random effects meta-regression: Combination of age, BMI & sample size as predictor/explanatory variable for the observed effect on emotion regulation*

```
 metareg SMD EDcode BMI, wsse (SE) graph

eta-regression                                    Number of obs  =        31
EML estimate of between-study variance            tau2           =    .02037
 residual variation due to heterogeneity          I-squared_res  =     0.00%
roportion of between-study variance explained     Adj R-squared  =    82.97%
oint test for all covariates                      Model F(2,28)  =      6.92
ith Knapp-Hartung modification                    Prob > F       =    0.0036
```

SMD	Coef.	Std. Err.	t	P>|t|	[95% Conf.	Interval]
EDcode	1.963957	.6859409	2.86	0.008	.5588709	3.369043
BMI	.1680242	.0482414	3.48	0.002	.0692061	.2668422
_cons	-8.393939	2.427191	-3.46	0.002	-13.36581	-3.422064

Figure S8. *Random effects meta-regression: Combination ED type & BMI as predictor/explanatory variable for the observed effect on emotion regulation*

```
 metareg SMD BMI EDcode Age, wsse (SE)

leta-regression                                   Number of obs  =        30
EML estimate of between-study variance            tau2           =    .03645
 residual variation due to heterogeneity          I-squared_res  =     0.00%
roportion of between-study variance explained     Adj R-squared  =    66.53%
oint test for all covariates                      Model F(3,26)  =      2.46
lith Knapp-Hartung modification                   Prob > F       =    0.0855
```

SMD	Coef.	Std. Err.	t	P>|t|	[95% Conf.	Interval]
BMI	.0675881	.1122726	0.60	0.552	-.1631915	.2983677
EDcode	.0433803	2.006829	0.02	0.983	-4.081716	4.168476
Age	-.0208993	.0247454	-0.84	0.406	-.0717641	.0299655
_cons	-1.681631	7.068215	-0.24	0.814	-16.21055	12.84729

Figure S9. *Random effects meta-regression: Combination of BMI, ED type & age as predictor/explanatory variable for the observed effect on emotion regulation*

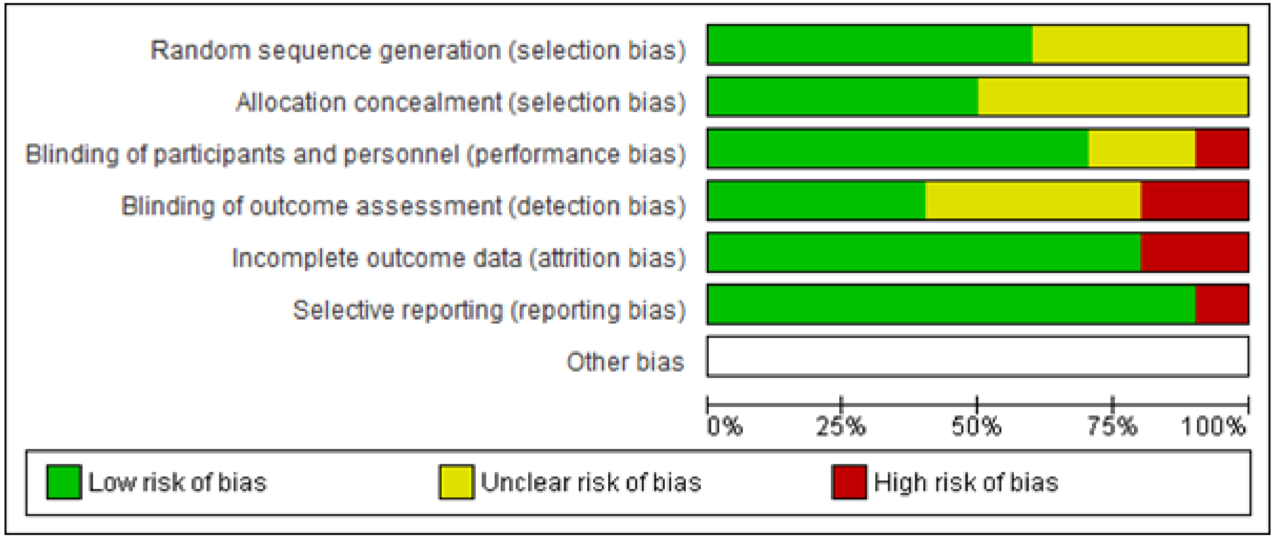

Figure S10. *Quality assessment: an overview by bias domain*

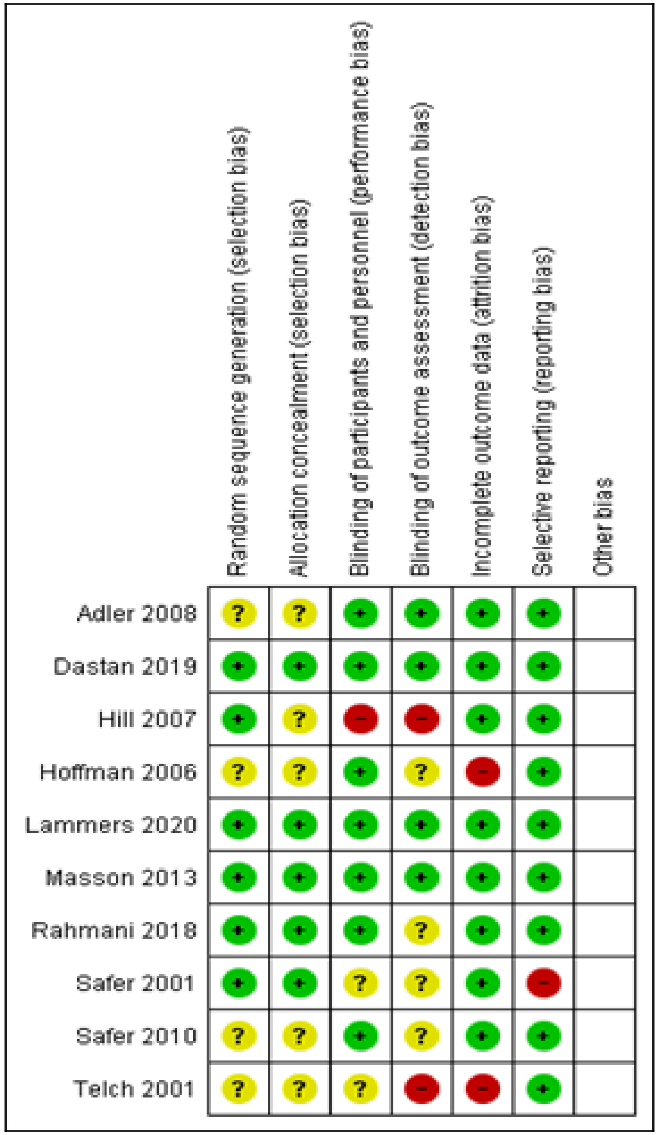

Figure S11. *Quality assessment: an overview by study*

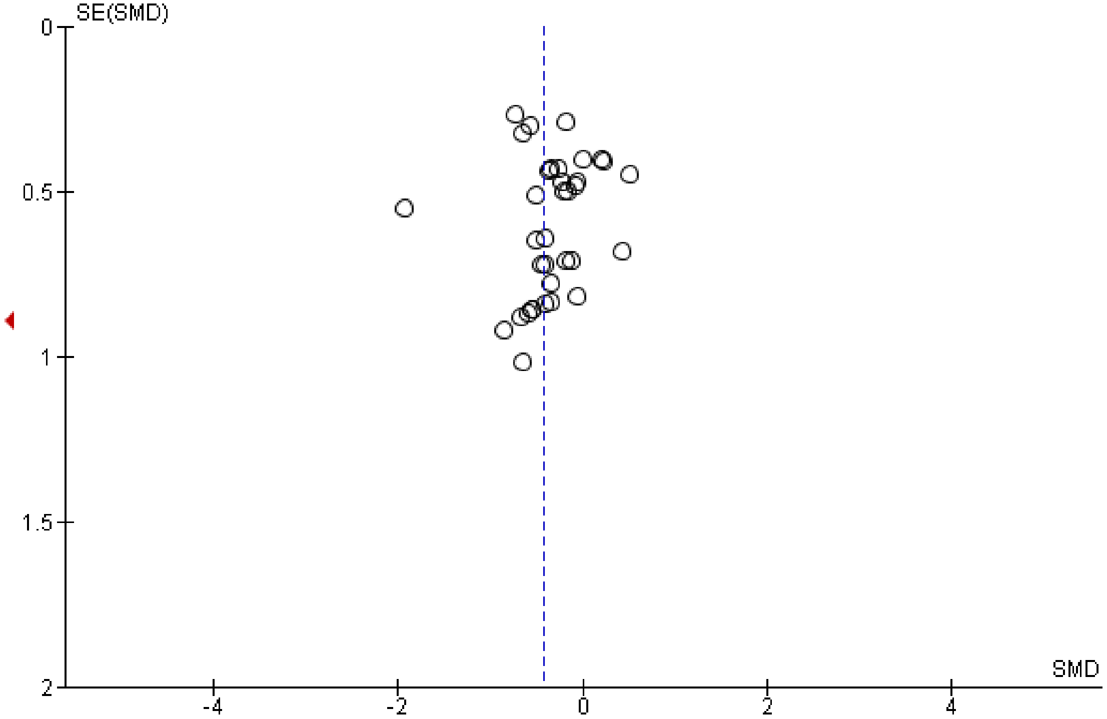

Figure S12. *Funnel plot: overall emotion regulation*

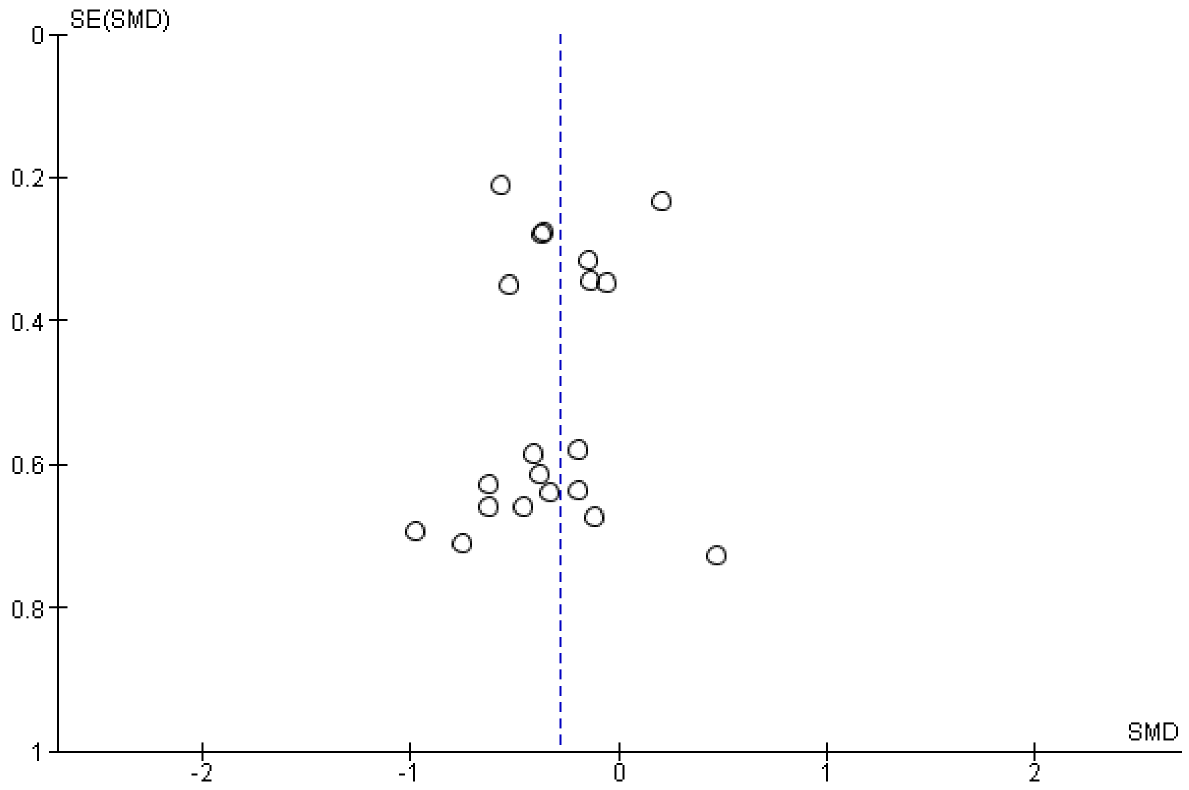

Figure S13. *Funnel plot: depressive symptoms*

2. Supplementary tables

Table S1. *Search terms used in the systematic search*

Query	Results
("anorexi*" OR "anorexia nervosa" OR "bulimi*" OR "bulimia nervosa" OR "eating disorder*" OR "binge-eating disorder" OR “feeding disorder” OR “OSFED” OR "EDNOS" OR “UFED” OR “ARFID” OR “orthorexia”) AND ("dialectic behavior therapy" OR "dialectic behaviour therapy" OR "dialectical behavior therapy" OR "dialectical behaviour therapy" OR "dialectic behavioral therapy" OR "dialectic behavioural therapy" OR "dialectical behavioral therapy" OR "dialectical behavioural therapy")	535

Table S2. Subgroup analysis results by comparison, non-adjusted

Subgroup analysis AT vs WL results															
**Outcome**	**Number of studies**	**(n)**		**MD/SMD**		**95 % CI**		**p-value**		**Heterogeneity (I^2)**		**p-value for I^2**		**p-value for subgroup test**	**I^2 for subgroup test**
		AT	WL	AT	WL	AT	WL	AT	WL	AT	WL	AT	WL		
**Overall emotion regulation**	36 / 11	1592	685	-0.23	-0.78	[-0.46, 0.01]	[-1.14, -0.42]	0.06	<0.0001	80%	80%	<0.00001	<0.00001	0.01	84.70%
**Eating emotion regulation**	8	301	145	0.22	-0.6	[-0.13, 0.57]	[-0.93, -0.26]	0.22	0.0005	52%	0%	0.1	0.82	0.001	90.80%
**General emotion regulation**	10 / 9	477	225	-0.16	-1.62	[-0.34, 0.02]	[-2.95, -0.28]	0.09	0.02	0%	94%	0.89	<0.00001	0.03	77.80%
**Depressive symptoms**	19 / 9	722	416	-0.24	-0.51	[-0.43, -0.05]	[-0.71, -0.31]	0.01	<0.0001	35%	0%	0.14	0.75	0.05	73.60%
**Objective binge episodes**	8	285	165	-0.13	-0.4	[-0.35, 0.09]	[-0.65, -0.14]	0.26	0.002	84%	81%	0.0003	0.001	0.12	59%
**Severity of symptoms**	8 / 7	192	240	-0.27	-1.24	[-0.87, 0.32]	[-1.91, -0.56]	0.37	0.0003	71%	82%	0.03	0.0002	0.04	77.30%
**BMI**	5	196	129	-0.33	-2.77	[-2.72, 2.06]	[-5.36, -0.18]	0.78	0.04	0%	56%	0.92	0.1	0.18	45.60%

Table S3. Subgroup analysis results by gender, non-adjusted

Subgroup analysis Females only (F) vs Females and males (F&M) results																
**Outcome**	**Number of studies**	**participants (n)**		**MD/SMD**		**95 % CI**		**p-value**		**Heterogeneity (I^2)**		**p-value for I^2**		**p-value for subgroup test**	**I^2 for subgroup test**
		F	F&M	F	F&M	F	F&M	F	F&M	F	F&M	F	F&M		
**Overall emotion regulation**	36 / 11	1170	1107	-0.72	-0.18	[-1.05, -0.38]	[-0.33, -0.03]	<0.0001	0.02	86%	34%	<0.0001	0.1	0.004	87.70%
**Eating emotion regulation**	8	254	192	-0.35	0.06	[-0.93, 0.24]	[-0.28, 0.40]	0.24	0.73	79%	21%	0.0008	0.28	0.42	0%
**General emotion regulation**	10 / 9	274	428	-1.51	-0.21	[-2.81, -0.21]	[-0.43, 0.01]	0.02	0.06	95%	24%	<0.0001	0.26	0.05	73.30%
**Depressive symptoms**	19 / 9	573	504	-0.44	-0.18	[-0.61, -0.27]	[-0.43, 0.08]	<0.0001	0.17	0%	47%	0.89	0.08	0.1	64.10%
**Objective binge episodes**	8	105	345	-0.45	-0.14	[-0.79, -0.10]	[-0.31, 0.03]	0.01	0.12	86%	81%	0.0007	0.0003	0.12	59%
**Severity of symptoms**	8 / 7	180	252	-1.35	-0.43	[-2.25, -0.46]	[-0.97, 0.10]	0.003	0.11	86%	74%	0.0001	0.009	0.08	66.60%
**BMI**	6	129	270	-2.77	-0.97	[-5.36, -0.18]	[-2.83, 0.88]	0.04	0.31	56%	0%	0.1	0.71	0.27	18.50%

Table S4. Subgroup analysis results by ED type, non-adjusted

Subgroup analysis BED vs miscellaneous EDs results															
**Outcome**	**Number of studies**	**participants (n)**		**MD/SMD**		**95 % CI**		**p-value**		**Heterogeneity (I^2)**		**p-value for I^2**		**p-value for subgroup test**	**I^2 for subgroup test**
		BED	Misc. EDs	BED	Misc. EDs	BED	Misc. EDs	BED	Misc. EDs	BED	Misc. EDs	BED	Misc. EDs		
**Overall emotion regulation**	36 / 11	1427	850	-0.4	-0.56	[-0.66, -0.14]	[-0.90, -0.22]	0.003	0.001	82%	81%	<0.0001	<0.00001	0.45	0%
**Eating emotion regulation**	8	276	170	-0.16	-0.21	[-0.52, 0.20]	[-1.09, 0.68]	0.39	0.65	50%	84%	0.09	0.002	0.92	0%
**General emotion regulation**	10 / 9	564	138	-0.84	-0.44	[-1.49, -0.19]	[-0.94, 0.05]	0.01	0.08	92%	36%	<0.0001	0.21	0.34	0%
**Depressive symptoms**	19 / 9	676	465	-0.22	-0.53	[-0.39, -0.05]	[-0.72, -0.34]	0.01	<0.0001	18%	0%	0.27	0.74	0.02	82.30%
**Objective binge episodes**	8	389	61	-0.15	-0.55	[-0.30, -0.01]	[-1.11, 0.01]	0.04	0.05	78%	91%	0.0005	0.0009	0.18	44.60%

Table S5. Subgroup analysis results by prioritization of ER, non-adjusted

Subgroup analysis primary ER (prim) vs. secondary ER (sec) results															
**Outcome**	**Number of studies**	**participants (n)**		**MD/SMD**		**95 % CI**		**p-value**		**Heterogeneity (I^2)**		**p-value for I^2**		**p-value for subgroup test**	**I^2 for subgroup test**
		Prim ER	Sec ER	Prim ER	Sec ER	Prim ER	Sec ER	Prim ER	Sec ERR	Prim ER	Sec ER	Prim ER	Sec ER		
**Overall emotion regulation**	36 / 11	1147	1130	-0.62	-0.33	[-1.04, -0.20]	[-0.46, -0.21]	0.004	<0.0001	91%	7%	<0.0001	0.37	0.2	39.50%
**Eating emotion regulation**	8	267	179	-0.06	-0.28	[-0.61, 0.49]	[-0.70, 0.15]	0.83	0.2	79%	39%	0.003	0.18	0.54	0%
**General emotion regulation**	10 / 9	347	355	-1.35	-0.23	[-2.48, -0.22]	[-0.44, -0.02]	0.02	0.03	95%	0%	<0.0001	0.49	0.06	72.50%
**Depressive symptoms**	19 / 9	419	613	-0.22	-0.4	[-0.43, -0.02]	[-0.60, -0.21]	0.03	<0.0001	0%	23%	0.58	0.22	0.2	38.20%
**BMI**	5	201	124	-2.26	-0.76	[-4.89, 0.36]	-3.60, 2.09]	0.09	0.6	67%	0%	0.05	0.57	0.45	0%

Table S6. Subgroup analysis results by comparison, adjusted

Subgroup analysis AT vs WL results (adjusted)																
**Outcome**	**Number of studies**	**Participants (n)**		**MD/SMD**		**95 % CI**		**p-value**		**Heterogeneity (I^2)**		**p-value for I^2**		**p-value for subgroup test**	**I^2 for subgroup test**
		AT	WL	AT	WL	AT	WL	AT	WL	AT	WL	AT	WL		
**Overall emotion regulation**	11	346	313	-0.2	-0.83	[-0.41, 0.01]	[-1.47, -0.20]	0.06	0.01	6%	77%	0.38	<0.00001	<0.01	86.50%
**General emotion regulation**	9	376	225	-0.16	-1.62	[-0.36, 0.04]	[-2.95, -0.28]	0.12	0.02	0%	94%	0.91	<0.01	0.03	77.60%
**Depressive symptoms**	9	394	145	-0.25	-0.39	[-0.45, 0.04]	[-0.73, 0.06]	0.02	0.02	5%	0%	0.4	0.99	0.46	0%
**Severity of symptoms**	7	192	196	-0.27	-1.14	[-0.87, 0.32]	[-1.70, -0.32]	0.37	<0.01	71%	66%	0.03	0.02	0.04	77%

Table S7. Subgroup analysis results by gender, adjusted

Subgroup analysis Females only vs Females and males results (adjusted)															
**Outcome**	**Number of studies**	**Participants (n)**		**MD/SMD**		**95 % CI**		**p-value**		**Heterogeneity (I^2)**		**p-value for I^2**		**p-value for subgroup test**	**I^2 for subgroup test**
		**Females**	**Females & males**	**Females**	**Females & males**	**Females**	**Females & males**	**Females**	**Females & males**	**Females**	**Females & males**	**Females**	**Females & males**		
**Overall emotion regulation**	11	346	312	-0.74	-0.24	[-1.29, -0.20]	[-0.46, -0.03]	0.008	0.03	77%	0%	<0.00001	0.77	0.07	69.70%
**General emotion regulation**	9	274	327	-1.51	-0.23	[-2.81, -0.21]	[-0.47, 0.02]	0.02	0.07	95%	20%	<0.01	0.29	0.06	72.40%
**Depressive symptoms**	9	254	285	-0.38	-0.19	[-0.63, -0.13]	[-0.47, 0.10]	<0.01	0.2	0%	24%	1	0.25	0.32	0%
**Severity of symptoms**	7	136	252	-1.26	-0.43	[-2.03, -0.50]	[-0.97, 0.10]	0.001	0.11	72%	74%	0.01	0.009	0.08	67.10%

Table S8. Subgroup analysis results by ED type, adjusted

Subgroup analysis BED vs other types of ED results																
**Outcome**	**Number of studies**	**Participants (n)**		**MD/SMD**		**95 % CI**		**p-value**		**Heterogeneity (I^2)**		**p-value for I^2**		**p-value for subgroup test**	**I^2 for subgroup test**
		**BED**	**Misc. EDs**	**BED**	**Misc. EDs**	**BED**	**Misc. EDs**	**BED**	**Misc. EDs**	**BED**	**Misc. EDs**	**BED**	**Misc. EDs**		
**Overall emotion regulation**	11	489	420	-0.48	-0.42	[-0.91, -0.05]	[-0.74, -0.10]	0.03	0.01	79%	0%	<0.00001	0.52	0.84	0%
**General emotion regulation**	9	431	170	-0.94	-0.4	[-1.75, -0.14]	[-0.71, -0.09]	0.02	0.01	93%	0%	<0.00001	0.45	0.22	34.2 %
**Depressive symptoms**	9	676	170	-0.22	-0.46	[-0.39, -0.05]	[-0.77, -0.15]	0.01	0.004	18%	0%	0.27	0.99	0.2	39.60%

Table S9. Subgroup analysis results by prioritization of ER, adjusted

Subgroup analysis primary ER vs. secondary ER results															
**Outcome**	**Number of studies**	**Participants (n)**		**MD/SMD**		**95 % CI**		**p-value**		**Heterogeneity (I^2)**		**p-value for I^2**		**p-value for subgroup test**	**I^2 for subgroup test**
		**Primary ER**	**Secondary ER**	**Primary ER**	**Secondary ER**	**Primary ER**	**Secondary ER**	**Primary ER**	**Secondary ER**	**Primary ER**	**Secondary ER**	**Primary ER**	**Secondary ER**		
**Overall emotion regulation**	10	387	272	-0.68	-0.3	[-1.27, -0.09]	[-0.54, -0.05]	0.02	0.02	85%	0%	<0.00001	1	0.83	0%
**General emotion regulation**	9	347	254	-1.35	-0.27	[-2.48, -0.22]	[-0.52, -0.02]	0.02	0.03	95%	0%	<0.0001	0.56	0.07	70.30%
**Depressive symptoms**	9	267	272	-0.17	-0.41	[-0.41, 0.08]	[-0.65, -0.16]	0.18	0.001	0%	0%	0.69	0.93	0.17	45.80%
